# COVID-19 in Africa: Catalyzing change for sustainable development

**DOI:** 10.1371/journal.pmed.1003869

**Published:** 2021-11-29

**Authors:** Salim S. Abdool Karim, Segenet Kelemu, Cheryl Baxter

**Affiliations:** 1 Centre for the AIDS Programme of Research in South Africa (CAPRISA), Durban, South Africa; 2 Department of Epidemiology, Columbia University, New York, New York, United States of America; 3 International Centre of Insect Physiology and Ecology (ICIPE), Nairobi, Kenya

## Abstract

Salim Abdool Karim, Segenet Kelemu and Cheryl Baxter discuss COVID-19 impacts and adaptations in Africa.

## Introduction

The Coronavirus Disease 2019 (COVID-19) pandemic has dramatically changed everyone’s lives, highlighting the fragility of systems providing public services, especially healthcare and food supply chains, and the need to build resilience, even in countries that are technologically and economically advanced. It is noteworthy how well most African countries have fared against all odds and defying doomsday predictions [1].

The first African case of COVID-19 was reported in Egypt on 14 February 2020. By October 2021, there were about 8.4 million cases and 216,000 deaths reported from Africa, accounting for about 3.5% and 4.4% of the world’s COVID-19 cases and deaths, respectively [2,3], despite comprising 15.4% of the world’s population. Notwithstanding the less resourced healthcare systems, the reported case fatality rate in Africa has been low at 2.6% [4]. Thus far, South Africa has been the most severely affected country in Africa, accounting for about a third of all cases on the continent.

There are several hypotheses for why Africa has not been as severely affected by COVID-19 compared to countries in Europe, or the United States [5]. While underreporting, undertesting, and suboptimal case detection may be contributing to the low rates, there is little indication that large numbers of symptomatic cases are being missed as there are few, if any, queues of patients with acute respiratory distress at hospitals in African countries. An important contributor to the lower cases in Africa is the youth bulge in its population pyramid [6]. Approximately 6.1% of the population in Africa is over 60 years [7] compared to 16.2% in the US. While seroprevalence surveys are yet to clarify whether Africa has had similar viral exposure to Europe, its younger population may explain its lower rates of clinical illness and deaths. In addition to Africa’s youth dividend, it is likely that there are other contributory factors that are yet to be demonstrated empirically. It has been speculated that prior infections with other coronaviruses may have created cross-reactive immunity [8,9] or that host genetics may be responsible due to unique host–pathogen coevolution in Africa [10].

Africa has experienced several challenges in responding to the COVID-19 pandemic, not least because it is simultaneously battling an unprecedented desert locust invasion of eastern Africa, fall armyworm invasion, malaria, HIV, and a multitude of existing health and agricultural concerns. The needs of the pandemic response come on top of significant pressures from political instability, corruption, and large migrant populations across the continent. Africa’s resource constraints place additional demands on innovation and adaptation for local circumstances. Rising to this challenge has not been easy, especially for the poorest countries, but has been accomplished in several settings across the continent.

### Creating continent-wide coordination for the COVID-19 response

The COVID-19 epidemic has created a common adversary that has served to bring African countries together in their response. The Africa Center for Disease Control (CDC) has been at the forefront of creating and coordinating the continent-wide approach that has been anchored in collaboration and solidarity. A Joint Africa Continental Strategy on COVID-19 was endorsed less than 6 weeks after the first confirmed case in Africa. Important successes of this strategy include the establishment of the Partnership to Accelerate COVID-19 Testing (PACT), which enabled Africa, initially sidelined when global demand for diagnostics rose, to increase the number of countries with testing capacity from 2 to 43 in 3 months, to procure more than 90 million test kits, and to train thousands of laboratory workers. The Africa Medical Supplies Platform pooled the procurement of critical medical supplies such as ventilators and personal protective equipment (PPE).

### How Africa pivoted its existing resources to respond to COVID-19

To meet local needs for masks, sanitizers, ventilators, and other medical supplies, African countries redirected existing manufacturing capabilities. For example, Kenya converted some textile manufacturing factories to mask and PPE assembly lines to become net exporters of these products. South Africa’s chemical industry produced sanitizer that was supplied to several other countries, and a household appliance manufacturer redirected its assembly line to produce several thousand ventilators.

Investments over the last 2 decades in local training, particularly epidemiological training, and research infrastructure through programs like the NIH’s Fogarty International Center and PEPFAR enabled some African countries to be better prepared and more responsive to the current COVID-19 epidemic. Several African countries utilized their community health worker programs [11] to carry out widespread screening and contact tracing in the community. Countries like Rwanda, Ghana, and South Africa creatively pivoted their testing infrastructure that had been established over many decades to address the Ebola, HIV, and tuberculosis epidemics to rapidly scale up Severe Acute Respiratory Syndrome Coronavirus 2 (SARS-CoV-2) testing [11] and implemented early pooled testing based on past experience of pooled testing for HIV. Ethiopian Airlines quickly adjusted by doubling its cargo flights capacity enabling it to distribute much-needed COVID-19 equipment, PPE, and other medications across the continent and beyond.

### Expansion of Africa’s health capacity to address COVID-19

Many African governments took decisive measures imposing restrictions early, drawing on lessons from their past experiences in dealing with epidemics and disasters. Ghana, Kenya, Liberia, Nigeria, Rwanda, Senegal, and South Africa are some of the African countries that prepared early and took measures including banning travel, closing borders, and closing schools, and several countries implemented early stay-at-home orders. Kenya and Ghana are ranked among the top 5 rated COVID-19 responders in the global COVID-19 response index [12]. The coronavirus pandemic served as an opportunity to strengthen healthcare capacity [13], surveillance, and laboratory capabilities. A major concern is vaccine nationalism, which has led to Africa being shunted to the back of the queue. Only 24 million of the 75 million doses committed by COVAX, the multinational vaccine procurement organization, up to May 2021 had arrived in Africa, due largely to manufacturers redirecting doses to local need. Concerningly, there are 10 countries in Africa that have not yet developed sufficient capacity to administer the COVID-19 vaccine that they have received.

### Changes to food security and availability

Recalling the 2007–2008 global food crisis and alerted by a potential negative impact on food security by COVID-19, the African Union brought early attention to the issue of food security. Among the measures taken was the exemption of local food markets and food distribution systems from COVID-19 lockdowns, classifying them as essential services. The flexibility and diversity of African food supply chains and food systems ranging from supermarkets, open markets, and small neighborhood stores to street vendors and roadside sales from car boots and pickup trucks served communities across countries. In some instances, taxi drivers and those who lost their jobs joined in the food supply chains.

Despite these initiatives, several African countries experienced a worsening of food security, particularly early in the epidemic. In South Africa, for example, informal traders were not able to operate during the initial lockdown, and 9 million children who usually receive their only daily meal through the National Schools Nutrition Programme were without a safety net while schools were closed [14]. Impressively, communities rallied through philanthropic initiatives by the nongovernmental organizations, faith-based organizations, and the private sector, together with government food parcels provided emergency food assistance. Given that food is vital, countries across Africa have developed a better appreciation for the need to do better in future epidemics to protect farmers, food producers, and those who are critical in the food supply chain.

### Looking to the future

Despite all the human sufferings, loss of life, economic and job losses, and unimaginable disruptions of day-to-day life, the pandemic also reminded the general public and governments around the world that investments in health, especially pandemic preparedness, pay off. Improved country-level planning for pandemic responses requires greater emphasis on “One Health.” Linkages created by COVID-19 between animal health and human health, biodiversity and climate change as well as ecosystems and socioeconomic conditions have provided a platform for Africa to address several of the key tenets of the African Union Agenda 2063 and the UN Sustainable Development Goals (SDGs) 2030.

COVID-19 has underscored the interplay of communicable and noncommunicable diseases [15], highlighting the need to strengthen the health systems to respond to this syndemic. This needs to go beyond physical infrastructure and healthcare worker training to fundamental changes that improve coordination and even integration between the private and public health sectors to achieve universal healthcare in pursuance of SDG 3.

In pursuit of SDG 2 on zero hunger, the COVID-19 epidemic presents an opportunity for countries to critically examine their food production systems and supply chains to make them more sustainable and resilient beyond pandemics to challenges such as invasive and destructive pests and climate change.

COVID-19 has also been an opportunity to strengthen the role of science to address not just health but economic and social challenges in Africa. It has also created the opportunity to revitalize research in Africa, elevating the role of scientists, especially in infectious diseases, and reawakened the 2007 African Union commitment to allocate 1% of GDP to research and development [16]. In establishing the most appropriate response at different stages to the COVID-19 epidemic, surveillance data, including big data, provided the basis for a rational approach to COVID-19. This increased transparency in the evidence guiding government decisions to control the spread of SARS-CoV-2 went a long way to build greater public trust in government.

While COVID-19 has taken Africa offtrack on its progress toward the SDGs, it has also created new opportunities to reimagine African society in its quest to achieve a better and more sustainable future for all on the continent.

## Abbreviations

CDC: Center for Disease Control and Prevention
COVID-19: Coronavirus Disease 2019
PACT: Partnership to Accelerate COVID-19 Testing
PPE: personal protective equipment
SARS-CoV-2: Severe Acute Respiratory Syndrome Coronavirus 2
SDG: Sustainable Development Goal
